# Application of weighted gene co-expression network analysis to explore the targets of treadmill training in rats with spinal cord injury

**DOI:** 10.3389/fneur.2026.1846393

**Published:** 2026-10-26

**Authors:** Haocong Zhang, Mingming Guo, Lingzhi Meng, Meihui Piao, Hailong Yu

**Affiliations:** Department of Orthopaedics, the General Hospital of Northern Theater Command, Shenyang, Liaoning, China

**Keywords:** co-expression network, miR-135a, protein-protein interaction network, regulatory network, spinal cord injury, treadmill training, weighted gene co-expression network analysis

## Abstract

**Background:**

Spinal cord injury (SCI) describes any damage to the spinal cord that results in a loss of function. With these injuries being commonly treated with subsequent treadmill training (TM), this study aimed to reveal the mechanisms facilitating TM-mediated rehabilitation following a SCI.

**Methods:**

Differentially expressed genes (DEGs) were identified and then subjected to a weighted gene co-expression network analysis to identify TM-associated modules. Co-expression network construction, functional annotation, protein–protein interaction (PPI) network, and transcription factor (TF)-miRNA-target regulatory network were then conducted. We established an SCI rat model and validated key RNAs using reverse transcription-quantitative polymerase chain reaction (RT-qPCR). Predicted interactions between miR-135a-5p and *JAK2* and *STAT1* were tested using dual-luciferase reporter assay.

**Results:**

Total 640 and 877 genes separately differentially expressed in SCI 8d + TM vs. SCI 8d and SCI 14d + TM vs. SCI 14d comparison groups were screened and merged as the DEGs, which were further narrowed to four TM-associated modules linked to the glutamatergic synapse and ribosome biogenesis pathways. PPI network evaluations identified v-Akt murine thymoma viral oncogene homolog 1 (AKT1), Janus kinase 2 (JAK2), tumor necrosis factor (TNF), and signal transducer and activator of transcription 1 (STAT1) as hub nodes. JAK2 and STAT1 were also shown to have interactions in module b. TF-miRNA-target regulatory network revealed that both *STAT1* and *JAK2* were targets of miR-135a. RT-qPCR results confirmed upregulation of *AKT1* and *STAT1* and downregulation of *JAK2*, *TNF* and miR-135a after TM. Dual-luciferase reporter assays revealed the direct binding of miR-135a-5p to *JAK2* and *STAT1*.

**Conclusion:**

These findings suggest that TM facilitates SCI recovery by regulating *AKT1*, *JAK2*, *TNF*, *STAT1* and miR-135a.

## Introduction

Spinal cord injury (SCI) is a term used to describe any damage to the spinal cord that results in temporary or permanent loss of any of its functions that includes muscle function, autonomic function, and sensation (1). SCI can be caused by traumatic factors (such as falls, gunshots, car accidents, and sports injuries) or nontraumatic factors (such as hypoperfusion, infection, and tumors) or both (2, 3). Location and severity of the SCI can also influence presentation of SCI in patients and manifest into a wide spectrum of symptoms ranging from minor pain, and numbness to paralysis and incontinence (4). SCI can also cause a variety of complications in patients, including pressure sores, muscle atrophy, respiratory problems, and infections (5). It is estimated that the global SCI incidence rate ranges from 236 to 4,187 per million (6).

Exercise has been shown to improve the muscular strength and physical capacity of patients with chronic SCI (7, 8). Treadmill training (TM) following an incomplete SCI may enhance lower-extremity motor function and gait recovery (9, 10). Exercise plays a critical role in the plasticity of the injured spinal cord by regulating various microRNAs (miRNAs) that target the phosphatase and tensin homologue (PTEN) and mammalian target of rapamycin (mTOR) signaling pathways (11). In animal models of SCI, appropriately dosed TM also promotes functional recovery, neuromuscular regeneration, and skeletal muscle plasticity (10). In addition, several studies suggest that the extracellular signal-regulated protein kinase 1 and 2 (ERK1/2) signaling pathway may function to transmit signals in SCI rats and promote the outgrowth of corticospinal tract axons following TM (12). TM following SCI also increases the endogenous expression of brain-derived neurotrophic factor (*BDNF*), which in turn helps to inhibit spasticity and allodynia by enhancing potassium-chloride cotransporter-2 (*KCC2*) expression (13–15). However, despite these observations, the underlying mechanisms facilitating TM-mediated recovery in SCI rats remain largely unknown.

In 2015, Baligand et al. (16) performed TM in a rat SCI model (moderate contusion SCI was produced using a NYU-MASCIS injury device) and then conducted an expression profiling analysis of the soleus muscles, which identified both the transforming growth factor beta (TGFβ)/SMAD family member 3 (SMAD3) signaling pathway and the bone morphogenetic protein (BMP) pathway as critical drivers of muscle reconstruction following SCI and TM. However, the group did not deeply explore the genes involved in the effects of TM on SCI rats. To address this aspect, we used a microarray dataset deposited by Baligand et al. (16) to complete a complex set of bioinformatics analyses designed to reveal the mechanisms facilitating TM-mediated rehabilitation following a SCI, and the key markers were verified by reverse transcription-quantitative polymerase chain reaction (RT-qPCR). This study will provide a better understanding of the far-reaching implications of posttraumatic healthcare following SCI.

## Methods

### Data source

Microarray dataset GSE45550 was downloaded from the Gene Expression Omnibus (GEO, http://www.ncbi.nlm.nih.gov/geo/) database and included the data from 24 rat soleus samples which comprised of 8 (SCI 8d, *n* = 6) and 14 (SCI 14d, *n* = 6) days after SCI, and 8 (SCI 8d + TM, *n* = 6) and 14 (SCI 14d + TM, *n* = 6) days after SCI with TM. All samples were evaluated using a GPL1355 [Rat230_2] Affymetrix Rat Genome 230 2.0 Array. In the original study of this dataset published by Baligand et al. (16), quadrupedal locomotor training was initiated on postoperative day 7 and consisted of 20-min treadmill stepping sessions. Training was performed three times in the SCI 8d + TM group and twice daily for 5 days in the SCI 14d + TM group. Manual body-weight support and assistance with plantar hindpaw placement were provided when necessary and gradually withdrawn as locomotor capability improved. However, the specific treadmill speed was not reported.

### Differential expression analysis

The gene expression profile data in CEL format was downloaded. The data were then preprocessed and normalized using the robust multi-array average (RMA) method in the oligo package^1^ (17) in R. RMA is a widely used technique for normalizing microarray data, which involves background correction, normalization, and summarization of probe-level data to obtain expression values for each gene (18). We used annotation platform information to match probes with their corresponding gene symbols. If multiple probes (multiple expression value) corresponded to a same gene, the average of these values was taken as the finally expression value of this gene. The normalized expression data were shown in Supplementary Table 1. We then used the limma package^2^ (19) in R to complete the differential expression analyses for SCI 8d + TM vs. SCI 8d and SCI 14d + TM vs. SCI 14d. Due to the inability to identify a sufficient number of differentially expressed genes (DEGs) after multiple testing corrections, *p*-values were not adjusted, and thus DEGs were screened with a cut-off value of *p*-value < 0.05, without an additional fold-change threshold.

### Weighted gene co-expression network analysis (WGCNA)

WGCNA is a systems biology algorithm designed to facilitate gene co-expression network construction using high-throughput mRNA expression data (20). Expression values of the DEGs across all 24 samples were used to construct the network. Sample consistency was assessed before calculating the adjacency and topological-overlap matrices. A soft-thresholding power was selected to approximate scale-free topology. Modules were identified using dynamic tree cutting with a minimum module size of 30 and were merged at a height threshold of 0.25. Module eigengenes were correlated with the TM phenotype, and key modules with |*r*| > 0.8 and *p* < 0.01 were obtained.

### Co-expression network construction and functional annotation

After identifying the TM-associated modules, we constructed co-expression networks around their nodes. In these networks, the edges represent co-expression relationships and the nodes represent critical genes, with each of these identified under the threshold of *p*-value < 0.05. Node genes were then subjected to Gene Ontology (GO)-based functional annotation using the DAVID tool^3^ (21). Moreover, we applied pre-ranked gene set enrichment analysis (GSEA) using both the full gene list and the list of DEGs as ranking strategies, independently validating the results at the 8-day and 14-day time points (SCI + TM versus SCI). Gene sets were defined by a custom GMT file containing the rat glutamatergic synapse pathway (rno04724) and ribosome biogenesis pathway (rno03008), and significance was assessed using an adjusted *p*-value of < 0.05.

### Protein–protein interaction (PPI) network and key gene identification

The genes involved in the co-expression networks were merged, and the PPIs among them were predicted using the STRING database^4^ (22). We applied a medium confidence of more than 0.4 and the PPI network was then constructed and visualized in Cytoscape software^5^ (23). Significant modules in the PPI network were identified using the MCODE plug-in^6^ (24) in Cytoscape software and the threshold score was set as > 5. Hub nodes with higher degrees in the PPI network were also screened and the expression profiles of these genes following SCI were evaluated using a short time-series expression miner (STEM) analysis. In addition, SCI-associated genes were retrieved from the Comparative Toxicogenomics Database (CTD; https://ctdbase.org/) and GeneCards^7^ using “spinal cord injury” as the search term. These genes were then intersected with the hub nodes in the PPI network to obtain key genes.

### Construction of transcription factor (TF)-miRNA-target regulatory network

We then used the Overrepresentation Enrichment Analysis (ORA) method from the WebGestal tool (25)^8^ to complete our miRNA- and TF-target predictions using the genes from the PPI network. The results with a *p*-value < 0.05 were selected and these target pairs were then integrated into a TF-miRNA-target regulatory network which was subsequently visualized using the Cytoscape software (23).

### Animals

Twenty adult Sprague Dawley rats (16 weeks of age, 220–250 g of weight) used in these evaluations were acquired from the animal experiment center at the General Hospital of Northern Theater Command of the Chinese People’s Liberation Army (Shenyang, China), and housed in separate cages at a temperature (22 °C ± 2 °C) and humidity (50 ± 10%) controlled environment under a 12 h/12 h light/dark cycle with free access to food and water. This study was approved by the Ethic Committees of the General Hospital of Northern Theater Command of Chinese People’s Liberation Army, Shenyang, China (No. 2022-015). All animal experiments comply with ARRIVE (Animal Research: Reporting of *In Vivo* Experiments) guidelines. This study was carried out in accordance with the NIH (National Research Council) Guide for the Care and Use of Laboratory Animals.

### SCI surgery and treatment

All rats were acclimatized to the environment for at least 1 week prior to the experiment. The rats were randomly divided into SCI surgery (*n* = 14) and control groups (*n* = 6). The SCI procedure was conducted as previously described (26). Briefly, 14 rats received prophylactic antibiotic treatment with ampicillin sodium (80 mg/kg) for 3 days before SCI surgery. The rats were anesthetized with isoflurane (3% for induction) and placed in a prone position on the operating table. Their chests and backs were shaved before undergoing a dorsal laminectomy at the thoracic vertebral T9 to expose the spinal cord. The spinal cord was then clamped with a vascular artery clamp for 1 min and then the tissue and blood vessels were removed. The incision was subsequently sutured. The rats were allowed to recover on the heat-controlled pad at 37 °C immediately after the surgery and were placed in a clean cage for observation. Rats were monitored until they were fully awake and able to move around. Ketopro-fen was injected subcutaneously in each animal (10 mg/mL) at 48 h post-surgery to relieve pain. The standards for successful generation of the rat SCI model were the presence of a contusion at the injury site, convulsions in both lower extremities, and spastic swaying of the tail. After SCI surgery, one rat was removed because it failed in modeling. Then the successfully model rats were randomly divided into four groups, including 8 days after SCI (SCI 8d), 14 days after SCI (SCI 14d), 8 days after SCI with TM (SCI 8d + TM), and 14 days after SCI with TM (SCI 14d + TM) (*n* = 3 each group). Then, rats in the SCI 8d + TM and SCI 14d + TM groups received quadrupedal locomotor training on postoperative day 7. Each training session consisted of 20 min of treadmill stepping at a speed of 10 m/min, with manual body-weight support provided when necessary. Among control groups, the rats in uninjured group (*n* = 3) were received no intervention, and the rats in uninjured+ TM group (*n* = 3) were received the same training as the other groups.

### RT-qPCR

All rats in different groups (SCI 8d, SCI 14d, SCI 8d + TM, SCI 14d + TM, uninjured and uninjured+ TM groups) were anesthetized with isoflurane (3% for induction) and sacrificed. The rat soleus samples were carefully separated from the T9 segment of the spinal cord of rats in these groups. Total RNA was extracted using TRIzol reagent via an extraction workflow comprising tissue homogenization, lysis, chloroform-mediated phase separation, isopropanol precipitation, 75% ethanol washing, and resuspension in RNase-free water. RNA concentration and purity were assessed spectrophotometrically, and samples with A260/A280 ratios of 1.8–2.1 were retained; RNA integrity was additionally assessed by agarose-gel electrophoresis. Then, cDNA was synthesized using Revert Aid First Strand cDNA Synthesis Kit (Thermo Scientific, Boston, USA). Then cDNA samples were processed into RT-qPCR, and PCR amplification was conducted using the SYBR green method (Takara Bio, Inc.) utilizing the Bio-Rad IQTM5 Multicolor Real-time PCR Detection System (Bio-Rad Laboratories, Inc.). The reaction conditions were as follows: 95 °C for 30 s, then 35 cycles of 10 s at 95 °C, 35 s at 60 °C and 1 min at 72 °C. Relative expression was calculated using the 2^−ΔΔCt^ method. The mRNA levels were normalized to β-actin and miRNA levels to U6 snRNA. The sequences of primers are listed in Table 1.

**Table 1:** Primers used in RT-PCR.

Genes	Primers
AKT1-F	CTTTATTGGCTACAAGGAACGG
AKT1-R	CAGTCTGAATGGCGGTGGT
JAK2-F	TATGCTCCCGAATCCTTGAC
JAK2-R	TCTGCCCTTGTTTATCATTGC
TNF-F	TCAGCCTCTTCTCATTCCTGC
TNF-R	TTGGTGGTTTGCTACGACGTG
STAT1-F	CATGGAAATCAGACAGTACCT
STAT1-R	TCTGTACGGGATCTTCTTGGA
miR-135A-F	GCCGGTATGGCTTTTTATTCCT
miR-135A-R	AGTGCAGGGTCCGAGGTATT
β-actin-F	TGTCACCAACTGGGACGATA
β-actin-R	GGGGTGTTGAAGGTCTCAAA
U6-F	ATTGGAACGATACAGAGAAGATT
U6-R	GGAACGCTTCACGAATTTG

### Dual-luciferase reporter assay

The predicted miR-135a-5p-binding sequences within the wild-type 3′ untranslated regions (3′UTRs) of human JAK2 and STAT1, together with the corresponding mutant sequences containing substitutions in the miR-135a-5p seed-binding sites, were separately cloned into the pmirGLO dual-luciferase reporter vectors. Human embryonic kidney 293T cells were maintained in Dulbecco’s modified Eagle’s medium (DMEM; Gibco, USA) supplemented with 10% fetal bovine serum (Gibco, USA) and 1% penicillin–streptomycin (Gibco, USA) at 37 °C in a humidified incubator containing 5% CO₂. Cells were seeded into 24-well plates and cultured overnight. At approximately 60% confluence, cells were co-transfected with the indicated reporter vectors and either the hsa-miR-135a-5p mimic or the corresponding negative control using Lipofectamine 2000 per well (Invitrogen/Thermo Fisher Scientific, USA) in Opti-MEM reduced-serum medium (Gibco, USA). Luciferase activities were subsequently measured using a Dual-Luciferase Reporter Assay Kit (Beyotime Biotechnology, China). Firefly luciferase activity was normalized to Renilla luciferase activity.

### Statistical analysis

Statistical analyses were performed using GraphPad Prism software (version 9.5.0, GraphPad Software Inc.; San Diego, CA, USA). Two-way analysis of variance (ANOVA), followed by Tukey’s multiple-comparisons test, was used to evaluate the effects of time and TM on key-gene expression. A two-sided Student’s t-test was used to compare the difference between two groups. Statistical significance was set at a *p*-value of < 0.05.

## Results

### Differential expression analysis

Our evaluations identified 640 (428 upregulated and 212 downregulated) and 877 (439 upregulated and 438 downregulated) DEGs in the SCI 8d + TM vs. SCI 8d and SCI 14d + TM vs. SCI 14d comparison groups, respectively (Supplementary Table 2). The union of the two sets of DEGs was included in the subsequent WGCNA. For overlapping genes in the two sets of DEGs, the average expression value of the gene across both sample groups were used as the final expression value for the subsequent WGCNA.

### WGCNA

We then determined the various parameters needed to produce a novel scale-free network distribution; these calculations included the determination of the adjacency matrix weighting parameter “power.” Once the value ranges for each parameter were established, we created a properly weighted scale-free distribution topology matrix. We then used the corresponding model and its statistics to create the selection graph used in our “power” calculations (Figure 1A). The higher the value of the square of correlation coefficient, the closer the network was to the scale-free network distribution. Thus, we selected our “power” value of 16 using the first instance, where the square of the correlation coefficient reached 0.9.

**Figure 1:**
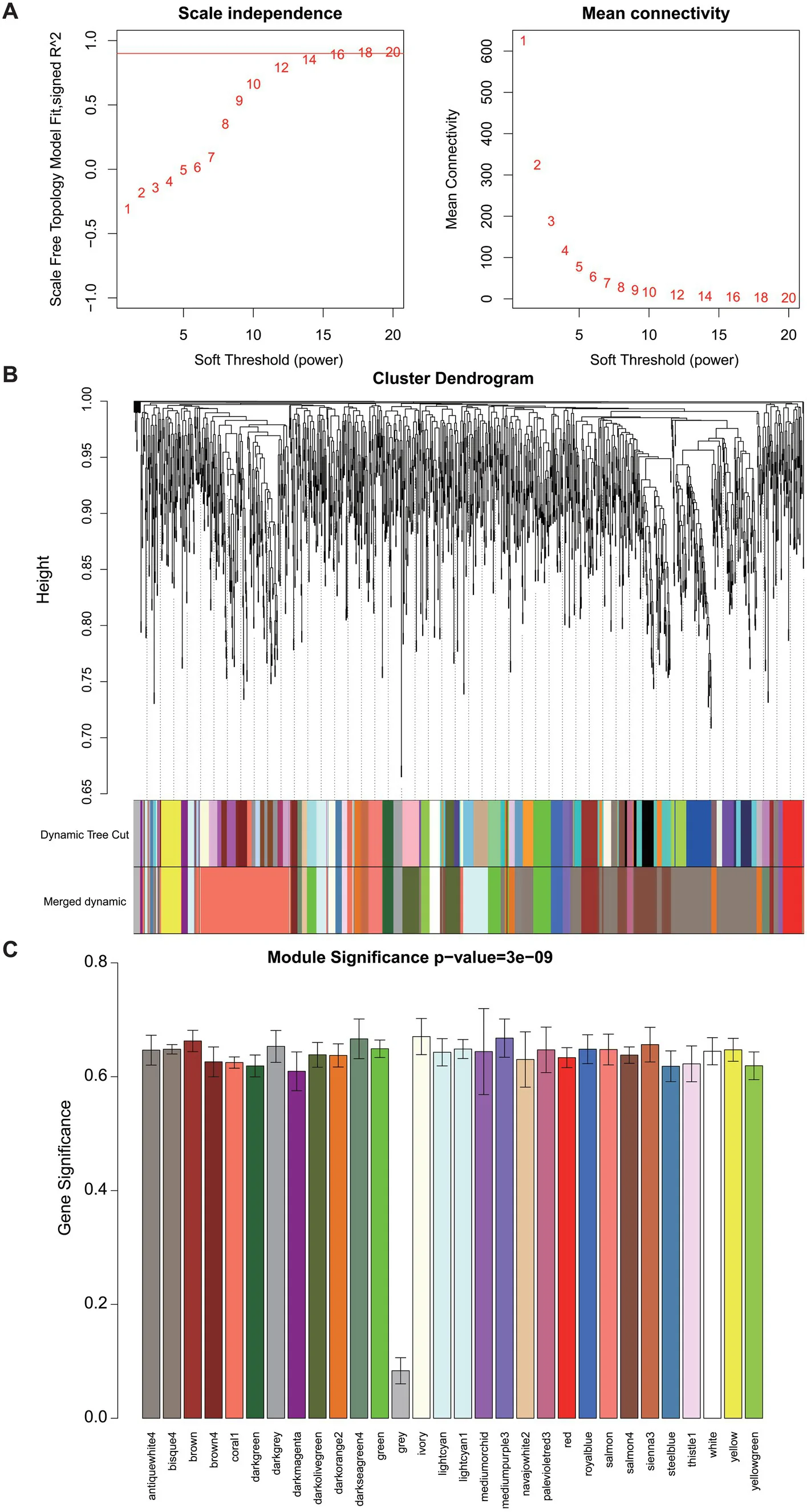
Weighted gene co-expression network analysis (WGCNA). **(A)** Selection graph used for evaluating the adjacency matrix weighting parameter, “power.” The red line represents the output when the square of the correlation coefficient reaches 0.9. **(B)** Cluster dendrogram describing the dissimilarity matrix, and the module distribution before and after merging DEGs. **(C)** Correlation diagram for locomotor training and module assignments.

We combined this “power” value with our algorithm and used this model to calculate the dissimilarity coefficients among each DEG. Subsequently, the cluster dendrogram was obtained by performing hierarchical clustering based on a dissimilarity matrix (Figure 1B). We then applied the standards used to develop hybrid dynamic shearing trees and set the minimum number of genes involved in each gene network module to 30. Once identified, we determined the feature vector “eigengenes” in each module and merged any modules with close clustering relationships into new larger groups (height was set at 0.25). Finally, the DEGs were divided into 30 modules, with those acting as outliers placed into the Grey module (Figure 1B).

We identified those modules that significantly correlated with the addition of TM and recorded the association between each module and specific sample feature vectors. These evaluations identified 7 modules with a |correlation coefficient| of > 0.8, and the top four modules were identified as follows: MElightcyan1, MEbisque4, MEdarkseagreen4, and MEgreen. Finally, analysis of the absolute gene significance (GS) values for each of these modules confirmed the reliability of our key module identification (Figure 1C).

### Co-expression network construction and functional annotation

We constructed co-expression networks for each node from modules MElightcyan1, MEgreen, MEbisque4, and MEdarkseagreen4 (Figure 2). GO functional annotation of the DEGs in each network revealed that module MElightcyan1 was closely associated with the regulation of synaptic transmission and the glutamatergic pathway (*p*-value = 2.11e-03), while MEbisque4 was associated with the negative regulation of actin filament polymerization (*p*-value = 6.10e-05). MEdarkseagreen4 was associated with calcium ion transmembrane transport (*p*-value = 7.71e-03) and MEgreen was closely linked to the negative regulation of transcription using DNA-templated responses (*p*-value = 0.018). These associations are summarized in Table 2.

**Figure 2:**
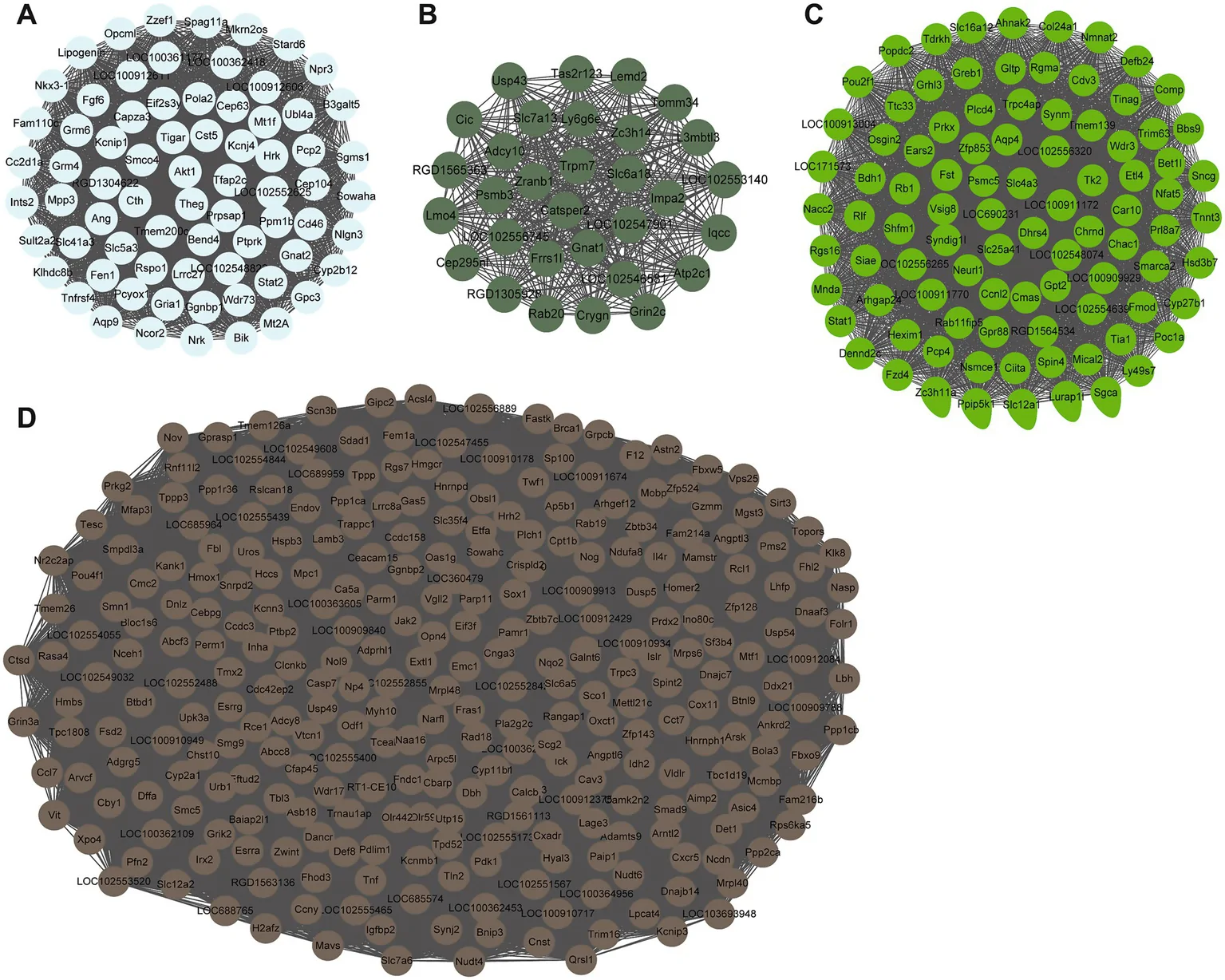
Co-expression networks outlining the nodes in the MElightcyan1 **(A)**, MEgreen **(B)**, MEbisque4 **(C)**, and MEdarkseagreen4 **(D)** modules.

**Table 2:** The gene ontology (GO) terms enriched for the differentially expressed genes (DEGs) in the co-expression networks for MElightcyan1, MEbisque4, MEdarkseagreen4 and MEgreen modules.

Category	Term	Count	p-value	Genes
MEbisque4	GO:0030837 ~ negative regulation of actin filament polymerization	5	0.000061	TWF1, PFN2, LOC100909840, FHOD3, KANK1
	GO:1900028 ~ negative regulation of ruffle assembly	3	0.00347	PFN2, LOC100909840, KANK1
	GO:2000378 ~ negative regulation of reactive oxygen species metabolic process	4	0.0058	BNIP3, PRDX2, BRCA1, SIRT3
	GO:1904707 ~ positive regulation of vascular smooth muscle cell proliferation	3	0.0122	TNF, JAK2, NQO2
	GO:0071243 ~ cellular response to arsenic-containing substance	3	0.0142	HMBS, HMOX1, UROS
	GO:0008219 ~ cell death	4	0.0146	GZMM, KLK8, HMOX1, BNIP3
	GO:0006644 ~ phospholipid metabolic process	4	0.0146	HMOX1, PLA2G2C, ANGPTL3, LPCAT4
	GO:0033160 ~ positive regulation of protein import into nucleus, translocation	3	0.0162	MAVS, JAK2, BRCA1
	GO:0030838 ~ positive regulation of actin filament polymerization	4	0.0253	CDC42EP2, PFN2, LOC100909840, BAIAP2L1
	GO:0042752 ~ regulation of circadian rhythm	4	0.0253	PPP1CA, HNRNPD, PPP1CB, OPN4
	GO:0050821 ~ protein stabilization	6	0.0289	CCT7, TESC, PFN2, LOC100909840, NAA16, DNLZ
	GO:0035556 ~ intracellular signal transduction	11	0.0302	RPS6KA5, ICK, ADCY8, HMOX1, DEF8, RGS7, JAK2, ARHGEF12, RASA4, ASB18, SCG2
	GO:0008630 ~ intrinsic apoptotic signaling pathway in response to DNA damage	4	0.0326	TNF, HMOX1, TOPORS, BRCA1
	GO:0007613 ~ memory	5	0.0334	NOG, KLK8, HRH2, DBH, NQO2
	GO:0007584 ~ response to nutrient	6	0.0351	HMGCR, OXCT1, ACSL4, IGFBP2, BRCA1, VLDLR
	GO:0043243 ~ positive regulation of protein complex disassembly	2	0.039	TNF, BNIP3
	GO:0070671 ~ response to interleukin-12	2	0.039	RIPK2, JAK2
	GO:0009725 ~ response to hormone	5	0.0419	HMBS, OXCT1, FHL2, ANGPTL3, VLDLR
	GO:0071361 ~ cellular response to ethanol	3	0.0456	PPP2CA, KCNMB1, CCL7
MEdarkseagreen4	GO:0070588 ~ calcium ion transmembrane transport	3	0.00771	CATSPER2, TRPM7, ATP2C1
MEgreen	GO:0045892 ~ negative regulation of transcription, DNA-templated	7	0.0181	CIITA, NACC2, PSMC5, HEXIM1, POU2F1, RB1, SMARCA2
	GO:0045944 ~ positive regulation of transcription from RNA polymerase II promoter	9	0.0472	CIITA, RGMA, POU2F1, NFAT5, GRHL3, RB1, STAT1, SMARCA2, CCNL2
MElightcyan1	GO:0051966 ~ regulation of synaptic transmission, glutamatergic	3	0.00211	GRM4, GRM6, NLGN3
	GO:0008285 ~ negative regulation of cell proliferation	6	0.00414	GRM4, PTPRK, CTH, GPC3, NKX3-1, NRK
	GO:0007196 ~ adenylate cyclase-inhibiting G-protein coupled glutamate receptor signaling pathway	2	0.0198	GRM4, GRM6
	GO:0032464 ~ positive regulation of protein homooligomerization	2	0.0254	HRK, BIK
	GO:0045926 ~ negative regulation of growth	2	0.0337	GPC3, MT2A

We then merged the nodes from the MElightcyan1, MEbisque4, MEdarkseagreen4, and MEgreen modules leaving a total of 492 genes (308 upregulated and 190 downregulated) for evaluation. GO analysis of these genes revealed that the majority of the 308 upregulated genes were associated with ion transmembrane transport (GO, *p*-value = 2.04e-04) and glutamatergic synapse (pathway, *p*-value = 7.03e-04). Meanwhile, the 190 downregulated genes were associated with neural tube closure (GO, *p*-value = 8.85e-03) and Ribosome biogenesis in eukaryotes (pathway, *p*-value = 3.14e-03) (Table 3). Preranked GSEA further revealed that the glutamatergic synapse gene set was enriched at day 8 (normalized enrichment score (NES) = 1.67, adjusted *p* = 0.0108). Neither glutamatergic synapse at day 14 nor ribosome biogenesis at either time point was significantly enriched.

**Table 3:** The Gene Ontology (GO) terms (A) and pathways (B) enriched for the merged nodes of the 4 modules.

Category	Term	Count	*p*-value	Genes
UP	GO:0034220 ~ ion transmembrane transport	8	0.000204	SLC12A1, AQP9, GRIA1, TRPM7, GRIK2, CLCNKB, GRIN3A, CNGA3
	GO:2000378 ~ negative regulation of reactive oxygen species metabolic process	5	0.000403	BNIP3, PRDX2, BRCA1, TIGAR, SIRT3
	GO:0030837 ~ negative regulation of actin filament polymerization	4	0.000119	TWF1, PFN2, LOC100909840, FHOD3
	GO:0071418 ~ cellular response to amine stimulus	3	0.00156	GRIA1, HMBS, UROS
	GO:0009725 ~ response to hormone	7	0.00162	AKT1, HMBS, OXCT1, FHL2, ANGPTL3, BDH1, VLDLR
	GO:1900028 ~ negative regulation of ruffle assembly	3	0.00321	PFN2, LOC100909840, ARHGAP24
	GO:0035235 ~ ionotropic glutamate receptor signaling pathway	4	0.0042	GRIA1, GRIN2C, GRIK2, GRIN3A
	GO:0006020 ~ inositol metabolic process	3	0.00425	SLC5A3, IMPA2, PPIP5K1
	GO:0001778 ~ plasma membrane repair	3	0.00542	CAV3, AHNAK2, MYH10
	GO:0005979 ~ regulation of glycogen biosynthetic process	3	0.00542	AKT1, PPP1CA, PPP1CB
	GO:0007613 ~ memory	6	0.00627	KLK8, TRPM7, HRH2, DBH, FEN1, NQO2
	GO:0006470 ~ protein dephosphorylation	6	0.014	PPP1CA, NCEH1, PPP2CA, PPM1B, PPP1CB, LOC100362453
	GO:0030838 ~ positive regulation of actin filament polymerization	4	0.0228	CDC42EP2, PFN2, LOC100909840, BAIAP2L1
	GO:0060416 ~ response to growth hormone	3	0.0286	AKT1, TRIM16, BDH1
	GO:0071456 ~ cellular response to hypoxia	6	0.0303	AKT1, NKX3-1, BNIP3, KCNMB1, TIGAR, VLDLR
	GO:0051966 ~ regulation of synaptic transmission, glutamatergic	3	0.0367	GRM4, GRM6, NLGN3
	GO:0032355 ~ response to estradiol	7	0.043	ESRRA, GRIA1, HMBS, DBH, BDH1, NCOR2, BRCA1
	GO:0098655 ~ cation transmembrane transport	4	0.0457	GRIN2C, SLC41A3, CHRND, CNGA3
	GO:0050804 ~ modulation of synaptic transmission	3	0.0486	GRIA1, GRIK2, NLGN3
DOWN	GO:0001843 ~ neural tube closure	5	0.0885	RGMA, NOG, SPINT2, LMO4, GRHL3
	GO:0043123 ~ positive regulation of I-kappaB kinase/NF-kappaB signaling	6	0.0105	CTH, TNF, ATP2C1, HMOX1, RIPK2, CC2D1A
	GO:0007283 ~ spermatogenesis	9	0.0158	POC1A, CATSPER2, TDRKH, RAD18, BIK, RB1, ADCY10, GGNBP2, GGNBP1
	GO:0006970 ~ response to osmotic stress	3	0.0209	LRRC8A, PCP4, NFAT5
	GO:0070671 ~ response to interleukin-12	2	0.0256	RIPK2, JAK2
	GO:1904037 ~ positive regulation of epithelial cell apoptotic process	2	0.0256	TIA1, JAK2
	GO:0030509 ~ BMP signaling pathway	4	0.0279	RGMA, NOG, FST, ZFP128
	GO:0045944 ~ positive regulation of transcription from RNA polymerase II promoter	16	0.0308	NOG, TNF, ZBTB7C, SOX1, CEBPG, LMO4, GRHL3, RB1, CCNL2, RPS6KA5, RGMA, MAMSTR, MTF1, H2AFZ, NFAT5, JAK2
	GO:0006513 ~ protein monoubiquitination	3	0.0309	RAD18, TOPORS, NEURL1
	GO:0051984 ~ positive regulation of chromosome segregation	2	0.0423	SMC5, RAD18
	GO:0008285 ~ negative regulation of cell proliferation	8	0.0433	PTPRK, CTH, TNF, CYP27B1, HMOX1, JAK2, RB1, NEURL1
	GO:0033209 ~ tumor necrosis factor-mediated signaling pathway	3	0.0464	TNF, JAK2, TNFRSF4

Category	Pathway	Count	*p*-value	Genes
UP	rno04724: Glutamatergic synapse	8	0.000703	GRM4, GRIA1, ADCY8, GRIN2C, GRIK2, GRM6, GRIN3A, HOMER2
	rno05031: Amphetamine addiction	5	0.00937	PPP1CA, GRIA1, GRIN2C, GRIN3A, PPP1CB
	rno04720: Long-term potentiation	5	0.00989	PPP1CA, GRIA1, ADCY8, GRIN2C, PPP1CB
	rno04024:cAMP signaling pathway	8	0.0132	AKT1, PPP1CA, GRIA1, ADCY8, GRIN2C, GRIN3A, CNGA3, PPP1CB
	rno04611: Platelet activation	6	0.0308	AKT1, PPP1CA, ADCY8, TLN2, PRKG2, PPP1CB
DOWN	rno03008: Ribosome biogenesis in eukaryotes	5	0.00314	RCL1, TBL3, UTP15, WDR3, FBL
	rno05152: Tuberculosis	6	0.0083	TNF, CYP27B1, CEBPG, RIPK2, JAK2, ARHGEF12
	rno03050: Proteasome	3	0.0419	PSMC5, PSMB3, SHFM1

### PPI network construction and key gene identification

The PPI network for the genes involved in the co-expression networks were constructed, which contained 249 nodes and 476 edges. In this network, v-Akt murine thymoma viral oncogene homolog 1 (AKT1) had the highest degree (degree = 35), indicating that AKT1 was a hub node. Further evaluation of this network facilitated the clear identification of module a (score = 7.714; 8 nodes and 27 edges) and B (score = 5.419; 8 nodes and 19 edges). Additionally, Janus kinase 2 (JAK2, degree = 18), tumor necrosis factor (TNF, degree = 14) and signal transducer and activator of transcription 1 (STAT1, degree = 14) all acted as nodes with high degrees within the PPI network, which were also interact with each other in module b (Figure 3). Besides, the SCI-related genes in CTD and GeneCards databases were searched, and the common genes in the two datasets were intersected with the four hub genes, and four overlapped genes were obtained, including *AKT1*, *JAK2*, *TNF* and *STAT1* (Figure 4A). In addition, *JAK2* and *TNF* presented with significantly decreasing expression while *AKT1* and *STAT1* presented with significantly increased expression in the 14-day SCI + TM group (*p*-value < 0.05; Figure 4B).

**Figure 3:**
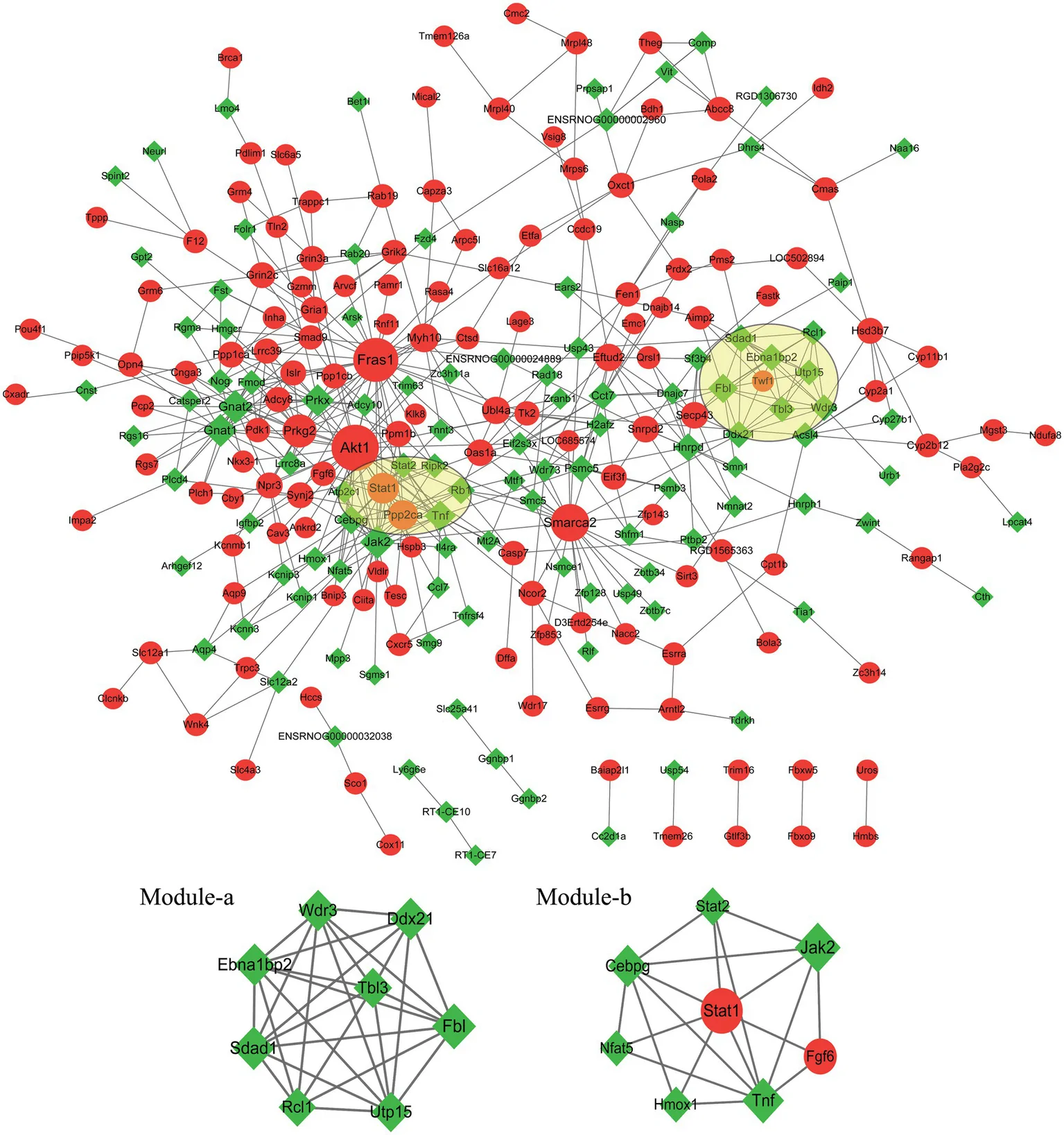
Protein–protein interaction (PPI) network highlighting the interactions between various gene products identified using the co-expression networks, and modules a and b in the PPI network. Red circles and green prisms represent upregulated and downregulated genes, respectively.

**Figure 4:**
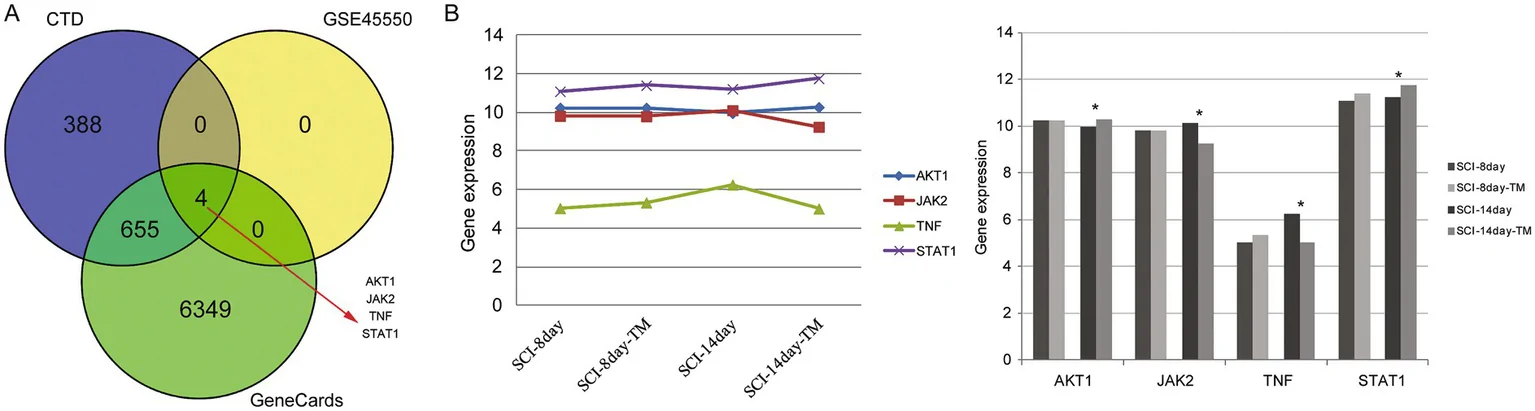
Identification of four key genes and analysis of their expression in different groups. **(A)** Venn diagram of key genes in Comparative Toxicogenomics Database (CTD), GeneCards, and GSE45550 databases. **(B)** Expression levels of key genes in different groups. *p-value < 0.05.

### Construction of the TF-miRNA-target regulatory network

Bioinformatics evaluations identified 14 TFs and 7 miRNAs, producing 310 TF-miRNA-target pairs (Supplementary Table 3). Subsequent visualization of this data revealed that both *STAT1* and *JAK2* were targeted by miR-135a (Figure 5).

**Figure 5:**
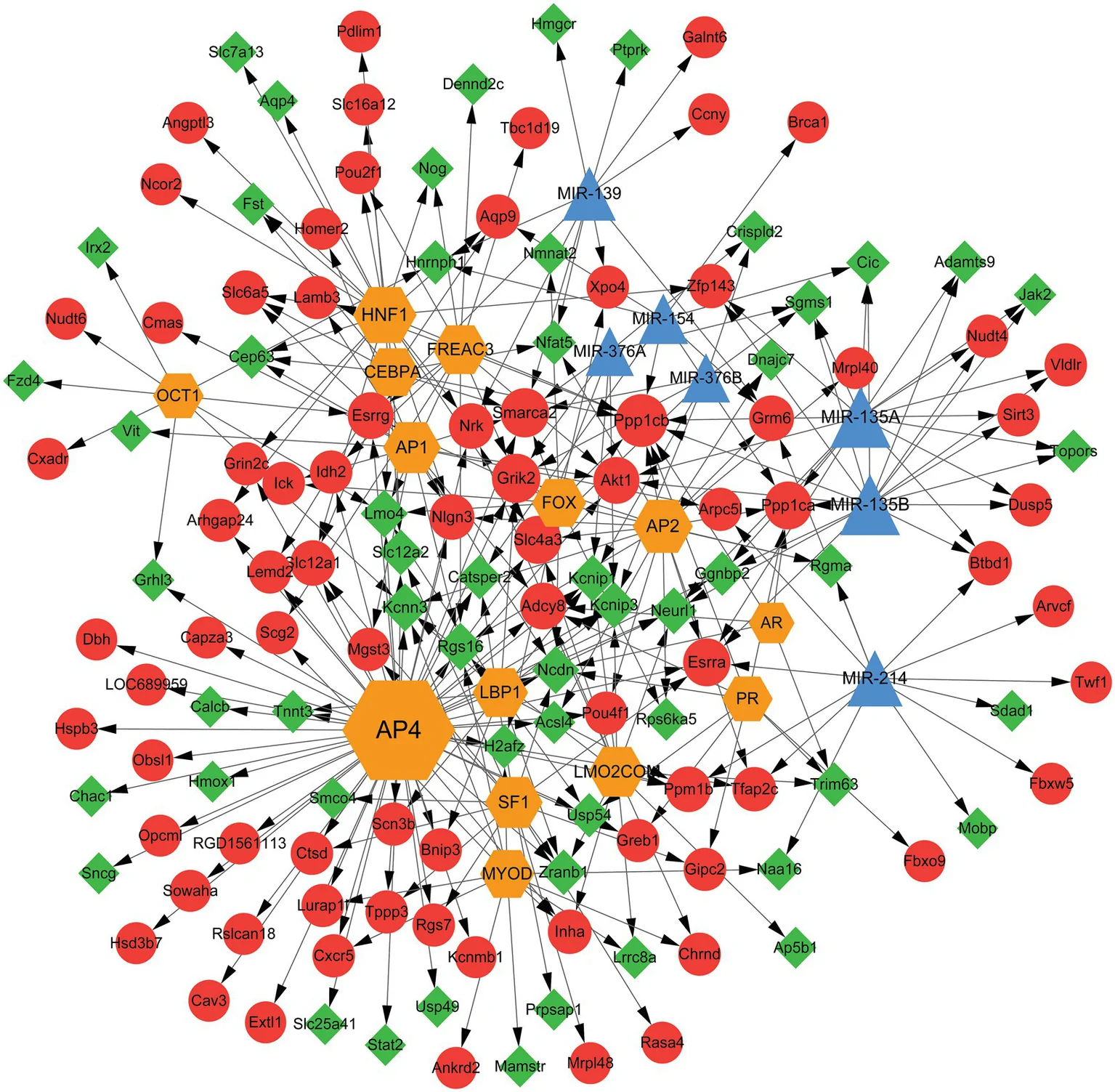
Transcription factor (TF)-miRNA-target regulatory network. Red circles, green prisms, blue triangles, and yellow hexagons represent upregulated genes, downregulated genes, miRNAs, and TFs, respectively. The arrows indicate the direction of regulation.

### RT-qPCR

We validated the key RNAs, *AKT1*, *JAK2*, *TNF*, *STAT1* and miR-135a, identified in our evaluations using RT-qPCR. The assays confirmed that both *AKT1* and *STAT1* were significantly upregulated in the SCI 8d + TM and SCI 14d + TM groups, while *JAK2*, *TNF* and miR-135a were significantly downregulated when compared to SCI 8d and SCI 14d groups, respectively (*p*-value < 0.05; Figure 6).

**Figure 6:**
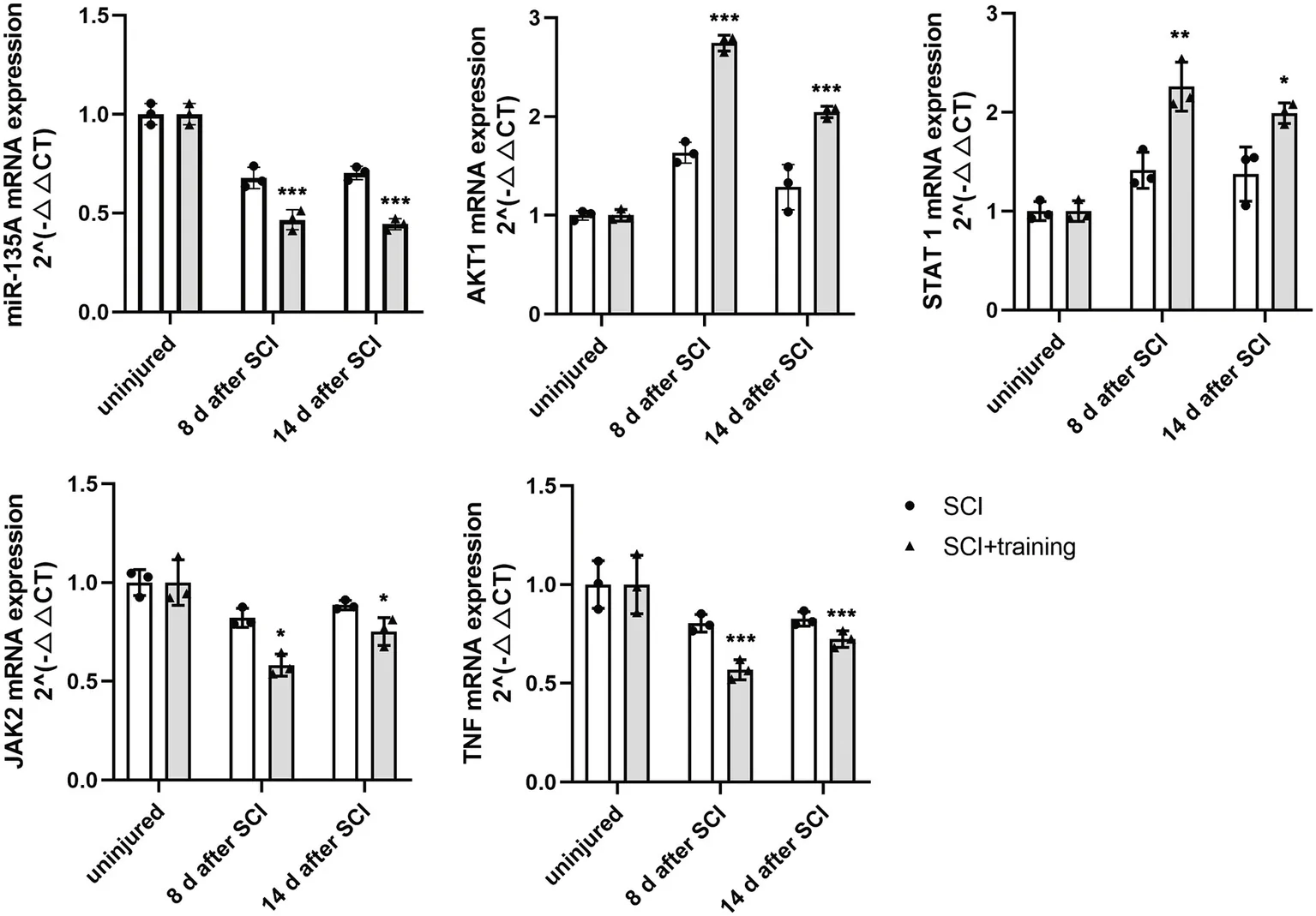
Reverse transcription-quantitative polymerase chain reaction (RT-qPCR) analysis of the five RNAs. Three rats were used in each group. SCI vs. SCI + training, *p-value <0.05, **p-value <0.01, and ***p-value <0.001.

### Dual-luciferase reporter assays

To validate the predicted interactions between miR-135a-5p and the 3’UTRs of *JAK*2 and *STAT1*, we performed dual-luciferase reporter assays. The results showed that the miR-135a-5p mimic significantly reduced the luciferase activities of the *JAK2* and *STAT1* wild-type 3’UTR reporters compared with the corresponding negative controls (*p* < 0.05), whereas no significant changes were observed for the mutant reporters. These findings support the direct binding of miR-135a-5p to the predicted sites within the *JAK2* and *STAT1* 3’UTRs (Figure 7).

**Figure 7:**
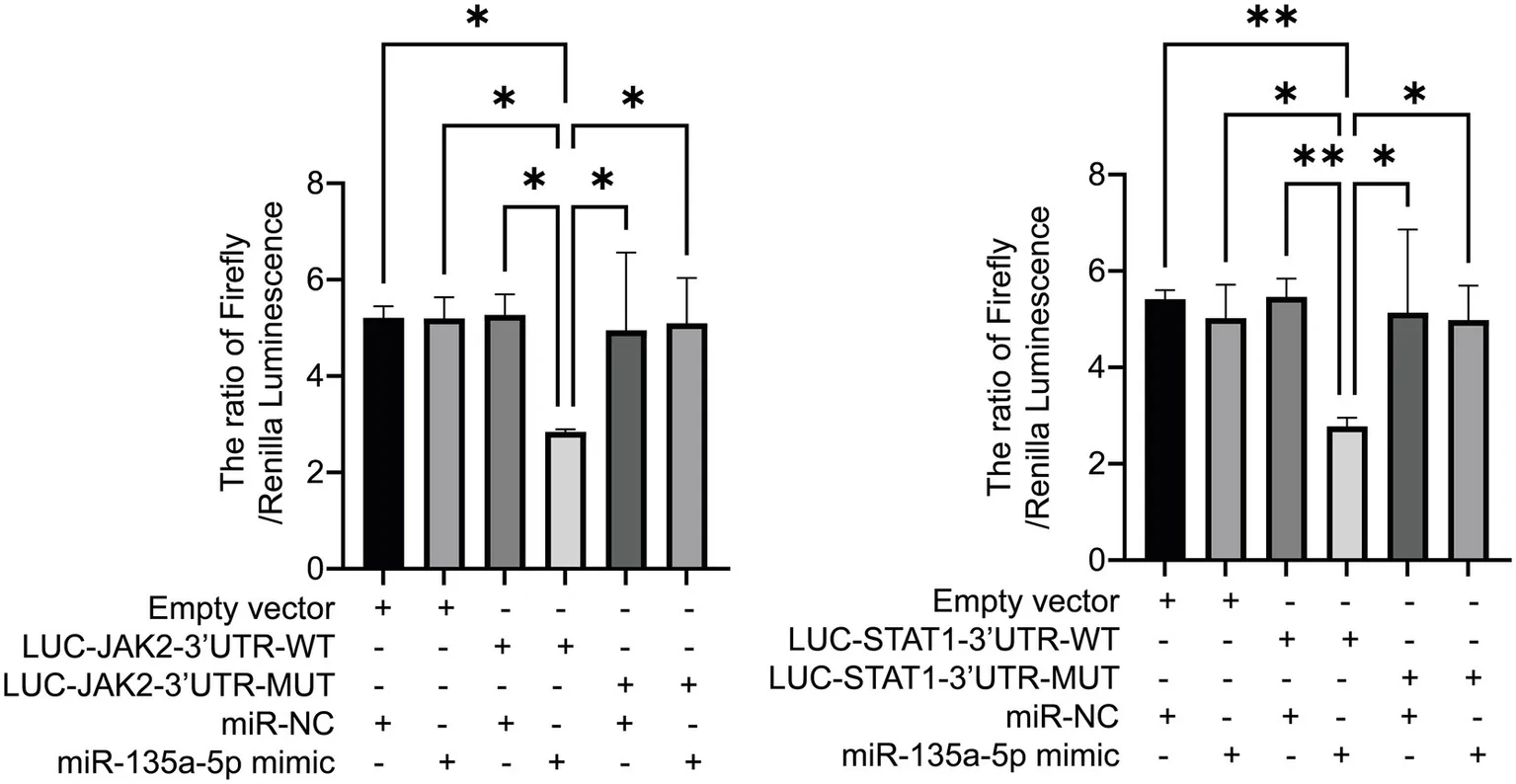
Dual-luciferase reporter assays revealed the direct binding of miR-135a-5p to the predicted sites within the 3′UTRs of *JAK2* and *STAT1*.

## Discussion

This study performed to reveal the mechanisms facilitating TM-mediated rehabilitation following a SCI. Our evaluations identified 640 DEGs in the SCI 8d + TM vs. SCI 8d and 877 DEGs in the SCI 14d + TM vs. SCI 14d comparison groups, which were combined to identify common DEGs for further evaluation. These combined DEGs were then used to construct a WGCNA network which included four TM-associated modules, MElightcyan1, MEbisque4, MEdarkseagreen4, and MEgreen. Co-expression network and functional analyses were conducted for the nodes of the four modules. In the PPI network, AKT1, JAK2, TNF, and STAT1 had high degrees. In addition, RT-qPCR results revealed that the *AKT1* and *STAT1* were significantly upregulated in SCI 8d + TM and SCI 14d + TM groups, while *JAK2* and *TNF* were significantly downregulated when compared to those in SCI 8d and SCI 14d groups, respectively. Besides, *AKT1*, *JAK2*, *TNF* and *STAT1* were the overlapped genes in CTD, GeneCards and GEO databases. Although *AKT1, JAK2, TNF* and *STAT1* are widely investigated as important pathway genes in many published papers, few studies reported *AKT1, JAK2, TNF* and *STAT1* in TM in SCI. Thus, this study provides theoretical basis and new insights for TM-mediated rehabilitation following a SCI.

Our findings agree with other studies which suggested that treadmill exercise upregulates neurotrophic factors via the phosphatidylinositol 3-kinase (PI3K)/Akt pathway, aiding in the functional recovery of locomotion by inhibiting apoptosis in the injured tissues of SCI rats (27, 28). The Akt pathway in spinal cord plays a protective role by mediating the endothelial nitric oxide (NO) pathway in SCI mice (29). Akt also regulates other biological processes in the muscle, including myoblast differentiation, protein synthesis, and metabolism. AKT1 has been shown to be the critical isoform associated with myoblast differentiation (30). Previous studies have revealed that moderate-intensity treadmill exercise activates cortical Akt/mTOR signaling and enhances neuroplasticity and functional recovery following SCI (31). *Chuanxiong Rhizoma* has been widely used in the SCI treatment, and AKT1 is the key target of *C. Rhizoma*-mediated anti-SCI activity (32). Tumor necrosis factor α (*TNFα*) induces apoptosis in oligodendrocytes and neurons, which may be regulated by NO and the dynamic regulation of inducible nitric oxide synthase (iNOS) (33). *TNFα* also plays critical roles in inflammatory pain and spinal cord synaptic plasticity via its regulation of tumor necrosis factor receptor 1 (*TNFR1*) and tumor necrosis factor receptor 2 (*TNFR2*) (34). A *TNFα* antagonist, etanercept, decreases the tissue damage associated with SCI, promoting myelin regeneration and enhancing locomotor function in SCI rats by inhibiting oligodendroglial and neuronal apoptosis (35). *TNFα* mRNA and protein expression are both elevated in the brains and spinal cords of mice with excitotoxic SCI, which may promote the development and progression of SCI (36). Therefore, this data supports our hypothesis that both *AKT1* and *TNF* were the targets of TM in SCI rats.

*STAT1* inhibition reduces histological damage and dyskinesia by regulating apoptosis and inflammatory response, making *STAT1* a promising therapeutic target for SCI rats (37, 38). *STAT1* and *STAT3* are both involved in neuronal cell death and survival, and their functional balance may affect the status of various neurons following a SCI (39). The JAK/STAT3 pathway is activated in the spinal microglia of SCI rats via interleukin-6 (IL-6) signaling and facilitates the development of nerve alteration-associated pain (40). However, the JAK2/STAT3 and mitogen-activated protein kinases (MAPK) pathways contribute to the regeneration and proliferation of injured spinal cord tissues, which may be utilized for the pharmacological intervention of SCI (41). In our evaluations, we noted that module b in our PPI network suggested that JAK2 and STAT1 also interact with each other in SCI rats, suggesting that these mediators may also impact pathological outcomes following a spinal cord injury. In addition, decrease in miRNA-135a may be correlated with increased GATA binding protein 3 (*GATA-3*) expression and both *GATA-3* and miR-135a may function to support exercise-induced immunosuppression (42). Wang et al. (43) reported that miR-135a-5p promotes the functional recovery of SCI via targeting *SP1* and *ROCK*. This finding is important as our data suggests that both *STAT1* and *JAK2* are also targets of miR-135a regulation, indicating that miR-135a expression might also correlate with SCI by targeting *STAT1* and *JAK2*.

Progressive skeletal muscle atrophy is one of the secondary physiological consequence of SCI (44). The development of muscle atrophy is governed by an imbalance between protein synthesis and degradation and is further influenced by apoptosis-related mechanisms (45). Because ribosome biogenesis and translational efficiency are major determinants of cellular protein-synthesis capacity, impaired ribosomal activity may contribute to the development of skeletal muscle atrophy (46). Ribosomal biogenesis is an important regulator of muscle growth and maintenance as the process directly impacts the translation capacity of the cell (47). In addition, Francolini et al. (48) revealed that when descending fibers of the spinal cord were surgically diverted to abdominal muscles via a nerve grafting procedure, supraspinal glutamatergic neurons can innervate these muscle fibers and restore motor function forcing these newly formed neuromuscular junctions to switch from a cholinergic-type to a glutamatergic-type synapse. In the present study, we found that the merged nodes of the four modules were primarily involved in glutamatergic synapse and ribosome biogenesis. Thus, we suspect that TM might facilitate SCI rehabilitation by affecting the glutamatergic synapse and ribosome biogenesis pathways.

From a therapeutic perspective, the identified candidate genes may be manipulated pharmacologically or genetically to promote recovery after SCI. Activation of AKT1-related signaling may enhance neuronal survival and axonal regeneration, as activation of the IGF-1R/Akt/mTOR pathway has been shown to promote corticospinal tract regeneration and motor recovery in experimental SCI (49). Conversely, inhibition of excessive JAK2/STAT-mediated signaling may alleviate neuroinflammation and neuronal apoptosis (50). TNF may also be targeted using neutralizing biologics such as etanercept, although the available evidence remains predominantly preclinical (51). Nevertheless, their therapeutic value requires further experimental validation.

However, despite the valuable insights provided by our findings, this study has several limitations. First, the use of unadjusted *P* < 0.05 to select DEGs increases the false-positive risk. Second, the discovery dataset and RT-qPCR samples were derived from soleus muscle. The observed signals may therefore reflect peripheral muscle adaptation to SCI and TM and cannot, by themselves, establish molecular repair within the spinal cord. Third, we limited our validation primarily to RNA expression and dual-luciferase reporter assays; assessment of protein abundance, phosphorylation, cell-specific localization, and the functional effects of *AKT1*, *JAK2*, *TNF*, *STAT1*, and miR-135a-5p require additional knock-down, overexpression, or pharmacological intervention studies. Fourth, only days 8 and 14 and male rats were evaluated, limiting temporal and sex generalizability. Although locomotor function was assessed by Basso-Beattie-Bresnahan locomotor rating scale scoring, individual behavioral outcomes were not correlated with molecular measurements. Finally, we did not integrate proteomic, phosphoproteomic, metabolic, or single-cell data in the present analysis. Collectively, larger-scale, sex-balanced, longitudinal, multi-omic studies incorporating functional experiments are needed in future research.

## Conclusion

In conclusion, our study identified *AKT1*, *JAK2*, *TNF*, *STAT1*, and miR-135a as candidate molecular factors associated with the responses to treadmill training after SCI. Taken together, the findings also suggest that TM may exert SCI rehabilitation effects via changes in the glutamatergic synapse and ribosome biogenesis pathways.

## Data Availability

The publicly available microarray dataset analyzed in this study is available in the Gene Expression Omnibus (GEO) under accession number GSE45550 (https://www.ncbi.nlm.nih.gov/geo/query/acc.cgi?acc=GSE45550). The original contributions presented in the study are included in the article/Supplementary material, further inquiries can be directed to the corresponding author.

## Footnotes

1
http://bioconductor.org/packages/release/bioc/html/oligo.html

2
http://www.bioconductor.org/packages/2.9/bioc/html/limma.html

3
https://david-d.ncifcrf.gov/

4
http://www.string-db.org/

5
http://apps.cytoscape.org

6
http://apps.cytoscape.org/apps/MCODE

7
https://www.genecards.org/

8
http://www.webgestalt.org/

## Glossary

SCI: Spinal cord injury
TM: treadmill training
DEGs: differentially expressed genes
WGCNA: weighted gene co-expression network analysis
GS: gene significance
GSEA: gene set enrichment analysis
NES: normalized enrichment score
PPI: Protein–protein interaction
TF: transcription factor
RT-qPCR: reverse transcription-quantitative polymerase chain reaction
JAK2: Janus kinase 2
TNF: tumor necrosis factor
STAT1: signal transducer and activator of transcription 1
miRNAs: microRNAs
PTEN: phosphatase and tensin homologue
mTOR: mammalian target of rapamycin
IGF1: insulin-like growth factor I
MRFs: myogenic regulatory factors
ERK1/2: extracellular signal-regulated protein kinase 1 and 2
BDNF: brain-derived neurotrophic factor
KCC2: potassium-chloride cotransporter-2
TGFβ: transforming growth factor beta
SMAD3: SMAD family member 3
BMP: bone morphogenetic protein
RMA: robust multi-array average
GO: Gene Ontology
STEM: short time-series expression miner
ORA: Overrepresentation Enrichment Analysis
PI3K: phosphatidylinositol 3 kinase
TNFα: Tumor necrosis factor α
iNOS: inducible nitric oxide synthase
TNFR1: tumor necrosis factor receptor 1
TNFR2: tumor necrosis factor receptor 2
IL-6: interleukin-6
MAPK: mitogen-activated protein kinases
GATA-3: GATA binding protein 3
